# Living in the twilight zone: a qualitative study on the experiences of patients with advanced cancer obtaining long-term response to immunotherapy or targeted therapy

**DOI:** 10.1007/s11764-022-01306-9

**Published:** 2022-12-10

**Authors:** Laura C. Zwanenburg, Karijn P. M. Suijkerbuijk, Sophie I. van Dongen, José J. Koldenhof, Anne S. van Roozendaal, Marije L. van der Lee, Melanie P. J. Schellekens

**Affiliations:** 1 Department of Scientific Research, Helen Dowling Institute: Centre for Psycho-Oncology, Bilthoven, The Netherlands; 2 Department of Medical and Clinical Psychology, Tilburg University School of Social and Behavioral Sciences, Tilburg, The Netherlands; 3 Department of Medical Oncology, University Medical Centre in Utrecht, Utrecht, The Netherlands; 4 Department of Public Health, Erasmus Medical Centre, Rotterdam, The Netherlands

**Keywords:** Psycho-oncology, Immunotherapy, Molecular targeted therapy, Lung neoplasms, Melanoma, Qualitative research

## Abstract

**Purpose:**

The introduction of immunotherapy and targeted therapy has drastically improved the life expectancy of patients with advanced cancer. Despite improved survival, obtaining long-term response can be highly distressing and comes with uncertainties that affect several life domains. The aim of this study is to gain a deeper understanding of long-term responders’ lived experiences with obtaining long-term response to immunotherapy or targeted therapy.

**Methods:**

We conducted an exploratory qualitative study using thematic data analysis. Semi-structured in-depth interviews were conducted with 17 patients with advanced melanoma or lung cancer who had a confirmed response to or long-term stable disease while on immunotherapy or targeted therapy.

**Results:**

Long-term responders are living in a twilight zone, where they neither feel like a patient, nor feel healthy. This impacts their self-image, interactions with their social environment, and feelings of uncertainty. Due to their uncertain life perspective, long-term responders are going back and forth between hope and despair, while they are longing for their ‘old’ life, several barriers, such as protective behavior of the social environment, force them to adjust to a life with cancer.

**Conclusion:**

Long-term responders are facing many challenges, such as searching for a renewed identity, dealing with ongoing uncertainty, and having to adapt to a new normal. This emphasizes the importance of providing this new patient group with tailored information and support.

**Implications for Cancer Survivors:**

Healthcare professionals can support patients by normalizing their feelings and providing space for varying emotions. Using patient-tailored scan frequencies could help temper fear of progression.

## Introduction

For stage IV melanoma or lung cancer patients, their diagnosis was previously accompanied by a dismal prognosis. Now, new treatment options such as immunotherapy and targeted therapy have drastically changed the life perspective of many patients with advanced (i.e., metastatic) cancer.

Before, the median overall survival of advanced melanoma was less than a year. After treatment with ipilimumab + nivolumab (immunotherapy), 46% of patients were still alive after 6.5 years [1]. Patients who respond to immunotherapy have a high chance of an ongoing response and becoming long-term survivors, even after stopping treatment [2, 3]. In advanced lung cancer, treatment with pembrolizumab (immunotherapy) increased the 5-year survival from approximately 6 to 25% [4, 5]. When treated with targeted therapy, the median survival of patients with metastatic lung cancer increased from 2.4 to 3.5 years [6]. Such long-lasting responses among advanced cancer patients have not been observed for other anticancer therapies. As a result, a new patient group has emerged: the long-term responders.

Despite improved survival rates, obtaining a long-term response in the face of an advanced cancer diagnosis can be highly distressing. Patients living long-term with metastatic cancer report several unique challenges related to coping with an uncertain prognosis, symptom management, financial burden, and care needs [7]. A survey study among advanced melanoma patients on immunotherapy or targeted therapy showed that the majority of patients reported anxiety when awaiting scan results (72% and 81%, respectively), feared melanoma recurrence or progression (81% and 86%, respectively), and feared death [8]. We know from earlier qualitative research that uncertainty about the disease trajectory (e.g., potential side effects and whether/when resistance to therapy will occur) is a major cause of distress in advanced melanoma and lung cancer patients on immunotherapy and targeted therapy [9–11]. Adjusting to and living with uncertainty seems to be a specific challenge for long-term responders and interferes with several aspects of daily life, such as relationships, work, and personal development.

Long-term responders form a new patient group within the larger group of those living with chronic cancer. Living with uncertainty is also a challenge mentioned by patients living with chronic cancer, but in contrast to long-term responders, they will presumably die with cancer and not because of cancer [12]. For long-term responders, the uncertainty that comes with the potential sudden occurrence of treatment resistance shapes every experience [9–11].

Although there is literature about patients living with cancer in general, literature remains sparse about this specific new and growing group of patients who obtain a long-term response [13, 14]. With a better understanding of how this patient group experiences obtaining a long-term response, healthcare professionals can provide care that better suits the patient’s situation. Such supportive care could prevent the development of psychopathology [15]. Our study aimed to gain a deeper understanding of long-term responders’ lived experiences with obtaining a long-term response to immunotherapy or targeted therapy.

## Material and methods

### Study design

We conducted an exploratory qualitative study among patients with advanced lung cancer and melanoma obtaining long-term response to immunotherapy and targeted therapy. Qualitative research is an interpretive, naturalistic approach to people’s worlds, giving voice to their own experiences and perceptions [16] and is therefore ideally suited to better understand long-term responders’ experiences.

### Recruitment and participants

Patients were recruited from April to June 2021 at Helen Dowling Institute (HDI), which is a mental healthcare center specialized in treating people suffering from clinical mental health problems because of their confrontation with cancer, and the outpatient clinics of University Medical Centre in Utrecht and Radboud University Medical Centre in Nijmegen, The Netherlands. We included patients diagnosed with stage IV lung cancer and stage IV melanoma with confirmed response to or long-term stable disease while on immunotherapy or targeted therapy (i.e., at least two consecutive scans confirmed that therapy is effective). By including both patients who have just learned that the treatment is effective and those who have been living with it for a long time, we hoped to gain insight into the psychological process long-term responders go through. We aimed for purposive sampling (i.e., we included patients who differed in gender, age, education level, time since diagnosis, and whether they had received psychological guidance) to obtain a broad perspective on patients’ lived experiences. Patients were at least 18 years of age and able to understand and use the Dutch language.

### Data collection

Sociodemographic and clinical characteristics were assessed via a short online questionnaire.

Semi-structured interviews were conducted by LZ, who has novice experience with interviewing for qualitative research, but who has been educated in various psychological interview techniques. During the interviews an interview guide was used (see Table 1), which was developed and pilot-tested with help from a long-term responder who was not included in this study. This interview guide contained questions about long-term responders’ experiences with the news that treatment is effective, their experiences with uncertainty regarding their disease and life perspective, and their experiences with their social environment. The interviews took place at a location of the patient’s preference (Helen Dowling Institute (*n* = 4), patient's residence (*n* = 3), or online via ZOOM (*n* = 11)), where only the researcher and participant were present, and lasted 41–69 min. After the audio-taped interview was transcribed verbatim, a summary was returned to the interviewee for a member-check. All participants indicated their answers had been correctly interpreted.

**Table 1:** Guide for semi-structured interviews

Topic	Questions
Opening	I would like to know how you are doing at the moment. Can you tell me something about how you are feeling today? • How are you doing physically? • How are you doing mentally?
Disease trajectory	I would like to know how the disease trajectory has been and how you experienced it. • Can you briefly tell me about your disease trajectory before you started immunotherapy/targeted therapy? • Can you go back to when the oncologist suggested to start immunotherapy/targeted therapy? • What were your thoughts about this therapy? • Can you briefly tell me about your disease trajectory after you started immunotherapy/targeted therapy? • What was it like when immunotherapy/targeted therapy turned out to be effective?
Living longer than initial expected when getting an advanced cancer diagnosis	Due to a good response to immunotherapy/targeted therapy, patients live longer, but with an uncertain prognosis. I would like to find out more about how you experience living longer. • How would you describe these experiences? What do you feel? What do you think? • What question should I ask to really understand how you experience this? • How did your social environment respond? What did you think of that? How did that affect you?
Living with an uncertain life perspective	I would like to know how you experience living with an uncertain life perspective • How would you describe these experiences? What do you feel? What do you think? • What question should I ask to really understand how you experience uncertainty? • How did your social environment respond? What did you think of that? How did that affect you? • To what extent do you make plans for the future?
Closing	• Is there anything else you'd like to discuss that we haven't already discussed? • What was it like taking part in this interview?

### Qualitative data analysis

Data were analyzed using inductive thematic analysis and using a subtle realism paradigm [17]. The language used by participants was seen as directly reflecting their experiences while acknowledging the inevitable effects of interpretation by the researchers during analysis [17]. Given this vulnerability to researcher interpretation, the multidisciplinary research team helped creating a broad view on long-term responder’s experiences. We followed the phases outlined by Braun and Clarke to structure data analysis (see Table 2 for extensive descriptions and examples) [18]. In the first and second within-case analysis phases, three researchers (LZ, AR, and MS) independently familiarized themselves with the transcripts and conducted initial open coding of the interviews using MAXQDA software [19]. Subsequently, they discussed the codes until reaching consensus. This led to a coding scheme on which the analysis of the following interviews was based. After the first five interviews were analyzed, one researcher (LZ) conducted additional interviews until data saturation was reached. Remaining interviews were coded by LZ. Codes that LZ was uncertain about were discussed with SD. Once all interviews were analyzed, two researchers (LZ and MS) organized the codes into potential topics according to the third cross-case analysis phase, which were checked with the interview data in the fourth phase. During the fifth phase, the multidisciplinary research team organized three meetings and grouped the codes and topics into themes. In the sixth and final phase, the manuscript was written.

**Table 2:** Qualitative research phases according to Braun and Clarke [18]

Phase	Description	Example
1. Familiarization	Listening to audio-tapes, transcribing interviews, and reading transcripts	-
2. Data coding	Coding interesting sentences	‘Stop buying new stuff’
3. Generating initial themes	Discussing codes, categorizing them into possible themes	‘Experiencing difficulties in making decisions because of the uncertain life perspective’
4. Reviewing and developing themes	Discussing themes, checking themes with data	
5. Refining, defining, & naming themes	Discussing and refining themes	‘Struggling to adapt to a life with cancer’
6. Writing the report	Writing the manuscript	-

**Table 3:** Sociodemographic and clinical characteristics of the 17 patients

	*n*	(%)
Age, M (SD)	56.4	13.0
Gender		
Men	6	35
Women	11	65
Education level		
Low	1	5.9
Intermediate	1	5.9
High	15	88.2
Working status		
Employed	8	47.1
Unemployed	1	5.9
Disabled	1	5.9
Sick	1	5.9
Retired	5	29.4
Volunteering	1	5.9
Relationship status		
Single	1	5.9
Married/Living together	14	82.4
Divorced	2	11.8
Cancer type		
Lung cancer	9	52.9
Melanoma	8	47.1
Time since diagnosis in months, M (SD)	38.3	26.4
Time since start current treatment in months, M (SD)*	27.7	18.5
Time since best response in months, M (SD)*	22.4	15.9
Treatment		
Immunotherapy*	13	76
Ipilimumab	1	
Ipilimumab & Nivolumab	1	
Nivolumab	4	
Pembrolizumab	6	
Targeted therapy	4	24
Afatinib	1	
Osimertinib	3	
Psychological guidance		
Before diagnosis	3	17.7
After diagnosis	9	52.9

^*^Data of 1 participant is missing

### Research team

All authors participated in data analysis and writing the manuscript. Their different backgrounds and perspectives facilitated a broad view on long-term responders’ experiences. All authors are female. LZ is a PhD-student with.

an MSc. degree in Health and Medical Psychology, who has completed an intensive course in qualitative research methods in healthcare. KS is a medical oncologist and JK is a nurse practitioner, both having extensive clinical experience with this patient group. SD is an epidemiologist and a post-doc researcher in psycho-oncology with prior experience with qualitative research in palliative care. AR is a student of the Research master Clinical and Health Psychology and research assistant. ML is a professor in medical psychology and healthcare psychologist, having extensive experience in treating this patient group. MS is behavioral scientist and works as a senior researcher in psycho-oncology. She is the principal investigator of the IMPRESS project of which this qualitative study is part.

### Ethics

This study was approved by the Ethical Review Board of Tilburg School of Social and Behavioral Sciences (RP487), which is a local review board. Since the study was not subject to the WMO, approval by a local committee was sufficient. The study was performed in line with the principles of the Declaration of Helsinki.

Healthcare professionals checked eligibility and provided patients with an information letter. The researcher (LZ) contacted interested patients by telephone to explain the study in more detail, ask for preliminary consent, and schedule the interview. All participants provided written informed consent prior participation.

## Results

### Participants

From April to July 2021, 21 patients were invited and 18 participated. Three patients declined because of logistic reasons (*n* = 1), expected emotional burden (*n* = 1), or unknown reason (*n* = 1). One patient was excluded from analysis after the interview, because she did not meet the inclusion criteria for treatment. Eventually, 17 participants were included in data analysis. Participants were mostly women (*n* = 11) and on average 56.4 years of age (see Table 3).

### Experiences of long-term responders

The analysis of the interview data resulted in three main themes: (1) *Twilight zone: neither feeling a patient, nor feeling healthy*; (2) *living with uncertainty: going back and forth between hope and despair*; and (3) *struggling to adapt to a life with cancer.*

### Theme 1: twilight zone: neither feeling a patient, nor feeling healthy

Participants described how they sometimes did not know which group they belonged to: neither to the healthy people who survived cancer, nor to the people dying from cancer. They felt as if they were living in a twilight zone, in between health and disease.

*“In the first year I felt like I belonged more with the dead than with the living. And then it got better and better. And I felt like I was a bit in between, you know. Now, it's so much better, like being one of the living again.”*—woman, 68 years, lung cancer, targeted therapy.

This feeling of ‘being in between’ appeared to change over time, shifting from feeling more of a patient to more of a healthy person. Feelings of uncertainty further fueled this feeling of being in between. Because next to happiness that there is an effective treatment available that helps prolong life, it was unknown whether and when therapy resistance would occur, creating great uncertainty.

*“When it seemed certain that I was going to die, it was super intense. Then it turned out that there’s therapy, which could make me live for another two or three years. That was very nice, yet also difficult, because it is also very uncertain. So, my son said “I hardly dare to say it, but I also think this is very difficult”. And then I thought yes, I get that. It’s the same for me. You really want certainty. You don't know what’s hanging over your head and when resistance will occur. It’s like death row without an execution date.”*—woman, 55 years, lung cancer, targeted therapy.

Despite treatment offering a better life-perspective, the threat of a forthcoming death was always present in the background. Long-term responders truly realized that life is finite when they got an advanced cancer diagnosis. Sometimes these thoughts of living a finite life seemed to fade for a while, but they resurfaced during moments of confrontation with the disease, for example, at time of a follow-up scan.

“*Of course, we’re all going to die. You could get hit by a truck and you’ll be dead. But when you hear that you will die, that’s such a big difference. It’s really hard to comprehend. Even now people sometimes say to me, well maybe I’ll be dead before you. Well, that’s fine, I'm just saying: you don't know, but I do know. I just feel I’m dying. And that’s the difference.”*—woman, 70 years, lung cancer, immunotherapy.

Although they are not yet dying from cancer, knowing you are going to die from cancer seemed to separate long-term responders from the healthy people. This also affected the identity of participants. Participants mentioned the loss of a carefree life and the feeling of no longer being considered a full member of society. Some participants were no longer able to work, while their work made them feel meaningful and was an important part of their identity.

“*That’s what I mean by not being equal anymore: people hang out with you because they feel pity, you know? I mean they do it out of love or out of friendship, but it's also because they feel sorry for you.”* – woman, 54 years, lung cancer, targeted therapy.

Feeling inferior to healthy people made it difficult for participants to let go of who they were before getting an advanced cancer diagnosis. Participants longed for their old self, who was a full member of society, making it difficult to accept the changes that came with obtaining a long-term response.

“*I would like to go back to my old self, but that doesn’t work of course. I have to go back to a new me, a new me that looks like the old me. That would be the best, that I can do again what I could before.”*—woman, 55 years, melanoma, immunotherapy.

Long-term responders realized that change is necessary or has already occurred. In particular, neither feeling like a patient, nor feeling healthy complicated the process of searching for their renewed identity. Not only the long-term responders themselves but also their social environment struggled with the extent to which the long-term responder is healthy or ill. According to participants, others often saw cancer as something you either are cured or die from. As a result, participants often felt misunderstood. They did not want to be seen as a patient, but also found it difficult if their disease and experiences were not recognized.

“*If someone tells me I’m doing really well, that [cancer] is surely over now, I think, “well, no, it’s not over at all”. But when someone starts on about how bad cancer is, then I think ‘stop it, it’s not like that at all, look at me, I’m here and I can do this and I can do that’. So, it’s always in between, more nuanced. One moment I can feel it this way and the next moment I can feel the opposite. So, others never really say it right. I think it’s very hard for people who want to support me, actually.”*—woman, 47 years, melanoma, immunotherapy.

As their own feelings and experiences regarding cancer changed quickly, they understood it was difficult for others to support them. In some cases, long-term responders mentioned feeling guilty towards their social environment. They felt guilty about not being as happy when others would expect them to be after good scan results. Feelings of guilt extended to how they changed as a person (e.g., more often feeling down, less enthusiastic about social events) and that their social environment also had to live and cope with uncertainty, possibly much longer than initially expected.

”*I feel sort of guilty towards the people around me. Because of what I do to them, even though I don’t do that deliberately. I know that, but still, their lives have also been turned upside down because of me.*”—woman, 55 years, lung cancer, targeted therapy.

Despite knowing it is not their fault, feelings of guilt and being misunderstood led long-term responders to keep their feelings or concerns about their illness for themselves. Some participants only shared their feelings with their close family. Participants stated that wanting to be seen as a person and not as a cancer patient also played a major role in the consideration whether or not to share their feelings.

“*At work, only my managerial director, one employee and my team manager whom I have very good contact with, know that I am ill. I deliberately chose to do it that way. And why? I just told you about my friends: when people think you have something going on, thinking you have cancer, ‘oh you’re sick, you don't get better’, you immediately get a label and people feel pity for you. That's the context, the paradigm I’m in. When they know, people will approach me differently in the sense that they will spare me and I don't want that. I'm not pathetic, I don't want to be pitied.”*—man, 44 years, melanoma, immunotherapy.

Long-term responders did not want others to feel sorry for them, because it made them feel inferior and put them in the patient role. These doubts about sharing their concerns, feelings or even their diagnosis further fueled their experience of shifting between being a patient and feeling healthy.

### Theme 2: living with uncertainty: going back and forth between hope and despair

Uncertainty in the face of obtaining a long-term response to immunotherapy or targeted therapy could be highly distressing. Participants mentioned not knowing what the future holds for them was a major struggle. They wondered whether it was worthwhile to make plans.

*“My oncologist says 'oh go live'. Literally: 'go live'. But how? With what future? Can I trust that I still have years to come? Yeah nobody knows, and neither do I. Making plans for the future can be difficult then*.” – man, 56 years, lung cancer, immunotherapy.

Regarding this uncertain future, some participants feared different doomsday scenarios. However, others conveyed that when things are uncertain, there is also room for hope. A positive mindset helped long-term responders to cope with uncertainty.

*“Then I said “if everything is uncertain, everything is still possible. So it can also go well.” And um yes, in this situation [coping with advanced cancer] you must also be able to see the positive side.”* –woman, 47 years, melanoma, immunotherapy.

When the future is uncertain, several scenarios are still possible. The results of the scan could be good and the treatment could be effective, possibly for a long time. Such a positive mindset helped participants to gain a sense of control in reaction to their uncertain life perspective. When they actively did something themselves, this increased their sense of control and made them believe they had some sort of influence on getting good results.

*“I have always done my very best to get fit again as quickly as possible, especially when I got out of the hospital. Then I'll at least have some spare if I get another setback.”* – man, 60 years, melanoma, immunotherapy.

Working on a good physical condition helped gain a sense of control, which could create more room for hope. A positive scan result also seemed to temporarily provide a sense of control, and lead to hope that the cancer would not progress, at least until the next check-up. Participants described seeing the time between scans as a fixed period of survival, in which they dared to make plans.

“*At the very beginning I got the scans every other month and then over time every three months and then every six months. In the beginning when it was every other month, that was my life window, my outlook for life. I also told myself it is one month, then we’ll see. That was manageable for me.”* -man, 56 years, lung cancer, immunotherapy.

Long-term responders felt safe until their next check-up, but an upcoming new scan would confront them with a lot of uncertainty again. While some participants hoped for a good scan result, other participants were driven to despair thinking progressive disease might become visible. This fear of progression seemed to gradually diminish with each positive scan, which resulted in more confidence and increased feelings of being in control.

*“The scans and the conversations afterwards are the moments when it really comes to the forefront again. Very present. This creates tension and uncertainty. However, this became less, every time it went well.”* -man, 56 years, lung cancer, immunotherapy.

While tension and uncertainty remained present, long-term responders slowly got used to frequent check-ups. Nevertheless, when time between two scans increased participants reported mixed feelings. On the one hand, they found this pleasant, because life became a little less dominated by the disease and it steered hope that cancer would not progress. On the other hand, it was also considered stressful, because this raised fear that new metastases would not be felt nor discovered in time and made participants feel less in control.

“*In fact, you don’t want all that hassle, having a scan and going to a hospital every time. Hey, I’m really not looking forward to that. It forces you to face reality again, like oh I have a scan again. On the other hand, it’s also nice to have that check. So it is a bit of a double feeling actually.”* – woman, 63 years, melanoma, immunotherapy.

Frequent control scans appeared to be both comforting as confronting for long-term responders. Participants were advised by their physicians to monitor their bodies. These self-exams were accompanied with mixed feelings. While monitoring their body, some participants got a sense of control over their fear for progression. Others described that, when feeling pain, they immediately perceived this pain as cancer-related and all hope seemed to vanish.

“*So I say “gosh, I've been in real pain here lately (points to belly). I probably have metastases again, the cancer has spread to the liver.” And… and that’s what happens. And then my sister says “your liver isn't there at all, it's in a completely different place.”*”—woman, 70 years, lung cancer, immunotherapy.

It appeared to be helpful when participants put the situation into perspective again and reappraise the pain, for example, with the help of their close others. Some patients had good hope that the cancer was under control when not seeing spots or feeling lumps while monitoring the body. However, other participants mentioned to struggle with monitoring their bodies. They explained they had lost trust in their body, especially when they had little to no complaints at time of cancer diagnosis.

“*And yes, it [concerns about cancer] is never gone of course, feeling light-hearted is gone, the confidence in my body was completely gone in the beginning. I was checking myself every day; do I see a spot again? Anyway, I still keep a very close eye on my body, and if I think something is going to change, I'll contact them [healthcare providers] anyway.”*—woman, 61 years, melanoma, immunotherapy.

Believing they were not able to see or feel whether something was wrong, made long-term responders feel out of control and could lead to excessive checking. This fueled their fear of progression.

### Theme 3: struggling to adapt to a life with cancer

Facing an uncertain life perspective could also change one’s view on life. Participants mentioned that they preferred going back to how their life was before getting an advanced cancer diagnosis. However, trying to go back to how life was before getting an advanced cancer diagnosis was complicated by several internal factors, such as difficulties with making decisions, loss of a carefree life, and doubts about one’s abilities (to work), and external factors, such as job changes or loss, protective behavior by close others and changed social contacts. First of all, participants mentioned worrying about making decisions, such as moving houses, changing jobs, or making (expensive) purchases.

“*There was a time when I stopped buying clothes. When I was in the store, I thought: I'm going to die anyway, why would I buy something?”*—woman, 33 years, melanoma, immunotherapy.

Engaging with long-lasting issues would confront participants with multiple questions: whether they would still be alive, whether it would be valuable and meaningful to invest time and energy, and whether they would benefit from their investment. Uncertainty and fear of progression always seemed to be present in the background. As a results participants no longer felt carefree, making it difficult to return to their “old” life.

*“I used to love to dance and have parties, but I don’t feel like it anymore. That's really gone.. yes. The carefree feeling that comes with partying, I don't have that anymore…”.* – woman, 54 years, lung cancer, targeted therapy.

While participants mentioned that they wanted to resume their ‘old’ lives again, they did put life on hold (e.g., by no longer buying new things or going to parties). A lack of self-confidence further prevented continuing their pre-cancer life. For example, participants indicated that returning to work or applying for a new job was often challenging due to doubts about their own abilities.

*“I also find it difficult to say convincingly that I am the very best candidate [for the job] when I don’t even feel confident that I am. Then I feel like I’m putting on a show.”* – woman, 47 years, melanoma, immunotherapy.

Apart from participants doubting their own abilities, employers also had their doubts. Participants mentioned their working contract was no longer extended or they were not hired due to their illness. This seemed to be a burden, especially since participants indicated that being able to go back to work was very important to them, as discussed in theme 1.

*“I also lost my job, and that was my outlet. They had to extend my contract, but what good is someone who is dying… I was supposed to get a permanent contract, that was promised verbally, but then I got the diagnosis, and then they said sorry…”* – woman, 33 years, melanoma, immunotherapy.

Next to their work life, their social life had also changed. Participants noticed it was difficult for their social environment to adapt to a life with cancer. Close others remained concerned and protective, despite participants doing well.

*“I'm fine now and let’s just get back on track as long as it goes well. Let’s pretend everything is “normal”. But every message [from the social environment], whether it’s a well-intentioned positive message like ‘Congratulations on the scan result, how great’ is another reminder that it is not normal.”* – man, 30 years, melanoma, immunotherapy.

As a result, participants were repeatedly confronted with their diagnosis, which made it more difficult to move on. Participants also experienced people coming and people going. While long-term responders received a lot of attention at time of diagnosis, when the disease process took longer than initially expected, several people did not stay in contact. Luckily, others remained present and, sometimes, new friendships were formed.

“*Well, you see some friendships really change, they are less fun, or more fun. For example, people who did not see you before, suddenly pull out all the stops. I always find that special, that all kinds of friendships suddenly arise, but also change again and um yes that has everything to do with the fact that I am sick.”*—woman, 54 years, lung cancer, targeted therapy.

These barriers within themselves, their work life, and social life made long-term responders realize it was not feasible to go back to their ‘old’ life and they needed to adapt to a new normal.

## Discussion

Our study provides insight into long-term responders’ lived experiences. Facing an uncertain life perspective made long-term responders feel like they were going back and forth between hope and despair and seemed to have a major impact on their feelings of being in control. When long-term responders felt in control, for example when getting physically fit, they also felt healthier, resulting in more room for hope. At moments of pain or when awaiting scan results, long-term responders felt more like a patient. They feared the cancer had progressed, leading to feelings of despair. Long-term responders were forced to adapt to a new normal in which uncertainty and feeling neither sick, nor healthy are omnipresent.

Our findings are in line with previous qualitative research among advanced lung cancer patients being treated with immunotherapy. In this study, the authors described how patients who survived longer than their original prognosis feel like living in limbo and describe this as a state that impacts life priorities, decision-making, experience of social support and health-information seeking behavior [20]. Another qualitative study among lung cancer patients who were treated with immunotherapy or targeted therapy described this as struggling to fit into the ‘sick’ role, especially when long-term responders experience no side effects of treatment [21]. Living in this limbo or twilight zone affects different life domains, including social relationships and self-image.

Similar to a recent study among advanced melanoma patients receiving immunotherapy, we found that social relationships of long-term responders are challenged [22]. Especially those who have been living for many years with an advanced cancer diagnosis experience that the social environment often does not know to what extent a long-term responder is healthy or ill. Others either under- or overestimate the long-term responder. For example, when the long-term responder looks healthy, others assume that the person also feels energetic, or when others learn someone has advanced cancer, they become overprotective [22, 23].

Adding to the current literature on long-term responders’ experiences [20–23], our findings show that long-term responders feel they have changed since time of diagnosis and notice changes in their self-image. For example, in the confrontation with the finiteness of life, they have lost a sort of carefree way of living. In addition to neither feeling like a patient, nor feeling healthy, these changes make long-term responders struggle with who they are. Accepting these changes and having to let go of who they were before diagnosis, further complicates the process of searching for a renewed identity.

Some of the described experiences, such as fear or anxiety regarding an uncertain life perspective, fear of disease progression, and fear of death also occur in various chronic diseases [24, 25]. Long-term responders are living long-term with advanced cancer and this may raise the question whether they can be considered as patients with chronic disease. A qualitative study among patients who are living with advanced lung cancer described that patients did not feel the term ‘chronic disease’ appealed to them, because they were living from scan to scan, ‘life was measured in 3 month increments’, and they did not expect to reach an old age such as many patients with chronic diseases as diabetes [26]. In particular, long-term responders differ from other chronic disease patients in that possible disease progression is not a slow decline, but might become visible at each check-up. Consequently, long-term responders often feel being tossed between hope and despair.

We know from earlier research that living with uncertainty causes great distress [27, 28]. The confrontation with the possibility of death and in particular uncertainty about how the cancer will develop makes patients vulnerable for developing psychopathology. Returning medical check-ups can cause feelings of uncertainty, contributing to vulnerability [29]. Such recurring control scans and ongoing uncertainty are part of the new normal for long-term responders, making them particularly vulnerable for developing psychopathology.

Together, our findings emphasize that due to the availability of new medical treatments, such as immunotherapy and targeted therapy, a new patient group has emerged that differs from patients surviving or dying from cancer. This new patient group has its own characteristics and challenges, most notably living with an uncertain life perspective for many months to even years, in which they are repeatedly confronted with possible disease progression [23]. Long-term responders report many unmet needs, including the need for more tailored information and more assistance with practical issues, such as finances, work and ongoing clinical trial participation [21, 22]. Thus, support that is specifically tailored to long-term responders is warranted [23].

### Strengths and limitations

This study only included Dutch participants with advanced melanoma or lung cancer, limiting the generalizability of the results to all advanced cancer patients obtaining a long-term response. On the other hand, a main aspect all long-term responders have in common is living with uncertainty, which could potentially determine long-term responders' experiences more than the type of cancer they are diagnosed with. By purposively sampling participants with different sociodemographic and clinical characteristics, we were able to map a wide variety of experiences. Moreover, data saturation was reached while encoding the data.

As in any qualitative study, it is vital to establish meta-positions and share preconceptions. While most of the authors work as researchers in the field of (psycho-)oncology, they have different backgrounds (i.e., oncologist, nurse practitioner, healthcare psychologist, epidemiologist, and behavioral scientist). All themes were extensively discussed within the multidisciplinary research team.

Most of the interviews were conducted online via ZOOM. Although literature suggests this can make it more difficult for patients to open up and share their vulnerabilities [30], long-term responders appeared to openly share their experiences and feelings with the interviewer and showed a wide variety of emotions during the interviews.

### Implications for clinical practice and research

The present study implies that long-term responders do not only experience happiness and relief when they obtain a good response to immunotherapy or targeted therapy, but also experience uncertainty and face ongoing challenges. It is important for healthcare professionals to become aware of the variety of emotions that people experience when obtaining a long-term response especially because this new group of long-term responders is expected to grow even faster in the forthcoming years [13]. Healthcare professionals can take a valuable step in supporting long-term responders by normalizing their feelings and providing space for their varying emotions. Frequent control scans, as well as the duration of time between scans, can have a lot of impact on long-term responders [31]. Discussing this impact and using patient-tailored scan frequencies, could possibly contribute to temper fear of progression and scan-related anxiety [32].

Following the recently published scoping review into psychosocial aspects of living long-term with advanced cancer and ongoing systemic treatment, we suggest more quantitative research into psychosocial outcomes of long-term responders is warranted, because literature about the consequences of obtaining a long-term response to immunotherapy or targeted therapy is currently still sparse [23]. In addition, future studies could also further explore what patients already do in day-to-day life to handle the uncertainty and ongoing challenges and what they experience as helpful in the face of these challenges. This could provide valuable input for what type of support and care would be most suitable for long-term responders.

## Conclusion

Effective treatment with immunotherapy or targeted therapy and the associated change in prognosis is good news for advanced cancer patients. However, this news comes with many challenges for long-term responders, such as struggling with searching for their renewed identity because they neither feel like a patient, nor feel healthy and being tossed between hope and despair due to ongoing uncertainty. Moreover long-term survivors experience recurring confrontations with their illness at medical check-ups. When they realize that it is not possible to go back to life as it was before the advanced cancer diagnosis, they have to adapt to a new normal. Our study emphasizes the importance of providing this new patient population tailored information and support.

## Data Availability

The data generated and/or analyzed in the current study are not available publicly as eligible patients were informed before start of the interviews that their data would be stored securely and confidentially.
